# Multiple Chronic Conditions Among Outpatient Pediatric Patients, Southeastern Michigan, 2008–2013

**DOI:** 10.5888/pcd12.140397

**Published:** 2015-02-12

**Authors:** Michael E. Rezaee, Martha Pollock

**Affiliations:** Author Affiliation: Martha Pollock, Oakland University William Beaumont School of Medicine, Rochester, Michigan.

## Abstract

Studies investigating the prevalence of multiple chronic conditions (MCCs) and their associated health care cost and use among pediatric populations have been limited. Among 14,404 pediatric patients receiving outpatient care in southeastern Michigan from 2008 through 2013, 82.1% had 0 chronic conditions, 16.2% had 1 chronic condition, and 1.6% had 2 or more chronic conditions. Greater numbers of chronic conditions significantly predicted outpatient cost (β = 581.7, *P* < .001), visit frequency (β = 9.1, *P* < .001), and days between appointments (β = −33.9, *P* < .001). Further study of MCCs among pediatric patients is needed given their increasing prevalence and their associated health care cost and use.

## Objective

Recent research concerning the epidemiology and cost implications of multiple chronic conditions (MCCs) has primarily centered on adult patients (1,2). The strong association of MCCs with age, health care use and cost, and reduced quality of life is an immense concern for the US health care system, which is preparing to care for a vast and aging baby-boomer population. Chronic disease is not unique to adults, however. Approximately 27% of children in the United States have a chronic condition and 1 in 15 have MCCs (3). Moreover, research indicates that the prevalence of chronic conditions is on the rise among pediatric patients (4,5). Studies investigating health care use and cost in this population have been limited. Most research has been conducted on children with special health care needs, which are often considered to include MCCs. However, research into children with special health care needs has primarily focused on children with disabilities, rather than broader pediatric populations with MCCs. The purpose of our study was to determine the prevalence and effect of MCCs among an outpatient population of children in Southeastern Michigan.

## Methods

We analyzed outpatient evaluation and management claims for patients under age 18 years and insured by the Beaumont Employee Health Plan (BEHP) to study MCC prevalence, cost, and use from 2008 through 2013. The BEHP is a regional health insurance provider serving Beaumont Health System employees (eg, physicians, nurses, clerical and facilities staff) and their families (spouses and children) in Southeastern Michigan. Beaumont Health System comprises 3 primary health care campuses (Royal Oak, Troy, Gross Pointe) and several satellite clinics and facilities in metropolitan Detroit. The BEHP provides health insurance coverage to approximately 30,000 people annually of whom about 7,000 are pediatric patients. The Beaumont Health System Research Institute for Human Investigation Committee (HIC) granted approval for this study (HIC no. 2014–051).

To determine MCC prevalence, the following 10 chronic conditions were identified by using *International Classification of Diseases, Ninth Revision, Clinical Modification* (ICD-9-CM) codes assigned to patients’ primary and secondary diagnoses on outpatient claims only: attention deficit disorder (ADD), asthma, autism, cancer, depression, diabetes, hyperlipidemia, hypertension, obesity, and substance abuse. Chronic conditions selected for this analysis represent a subset of prevalent and potentially preventable diseases specified by the Office of the Assistant Secretary for Health of the US Department of Health and Human Services for the purpose of standardizing MCC research initiatives (6). The conditions selected for the subset were those diseases that were deemed relevant to pediatric populations on the basis of the authors’ clinical judgment. Patients were organized into the following chronic condition categories: 0 chronic conditions, 1 chronic condition, and 2 or more chronic conditions (ie, MCCs).

Average outpatient cost, number of outpatient visits, and days between appointments were calculated for each chronic condition category for each year of the study. Cost was defined as the total dollar amount paid for outpatient services by BEHP for each patient during the study period. We examined the crude and adjusted relationship between MCCs and outpatient cost, visit frequency, and days between appointments by using linear regression, adjusted for age and sex. Using the year 2008 as a reference, adjusted odds ratios for MCC occurrence were calculated for each year of the study period using multiple logistic regression. Ten pediatric patients (<0.01%) were dropped from the analysis because of missing demographic or outcomes information.

## Results

The study population consisted of 14,404 pediatric patients with an average age of 7.6 years (standard deviation, 6.0 y). Among these patients, 82.1% (n = 9,709) had 0 chronic conditions, 16.2% (n = 2,333) had 1 chronic condition, and 1.6% (n = 230) had 2 or more chronic conditions (Table 1). Outpatient health care costs for pediatric patients with MCCs were almost 4 times that of pediatric patients with no chronic conditions ($1,682.80 vs $460.60). Similarly, pediatric MCC patients had significantly more outpatient visits (26.1 vs 8.3) and fewer days between appointments (90.2 vs 153.1) than patients without chronic conditions. Adjusted regression analyses revealed that greater numbers of chronic conditions significantly predicted outpatient cost (β = 581.7, *P* < .001), outpatient visit frequency (β = 9.1, *P* < .001), and days between appointments (β = −33.9, *P* < .001). Asthma (9%), ADD (5.4%), the combination of asthma and ADD (1%), depression (0.6%), and diabetes (0.4%) were the most prevalent chronic conditions in this population (Table 2). These chronic conditions accounted for 30% of the BEHP’s total pediatric outpatient cost.

**Table 1:** Prevalence and Outcomes by Number of Chronic Conditions for Children Under 18 Years of Age (n = 14,404) in the Beaumont Employee Health Plan, Southeastern Michigan, 2008–2013

No. of Chronic Conditions	Percentage of Pediatric Population With Conditions^a^	Average Outpatient Cost, $	Average No. of Outpatient Visits	Average No. Days Between Appointments
0	82.1	460.5	8.3	153.1
1	16.2	1,000.2	16.9	122.6
≥2	1.6	1,682.8	26.1	90.2

a Percentages do not add to 100 because of rounding.

**Table 2:** Prevalence and Associated Outpatient Cost for the Top 5 Chronic Conditions Among Children Under 18 Years of Age (n = 14,404) in the Beaumont Employee Health Plan, Southeastern Michigan, 2008–2013

Chronic Condition	No. of Pediatric Patients	Percentage of Pediatric Population With Conditions	Total Outpatient Cost, $	Total Outpatient Cost, %
Asthma	1,298	9.0	1,394,594	17.0
ADD	780	5.4	671,444	8.2
Asthma and ADD	148	1.0	250,451	3.1
Depression	87	0.6	71,531	0.8
Diabetes	60	0.4	65,234	0.7

Abbreviation: ADD, attention deficit disorder.

From 2008 through 2013, the proportion of pediatric patients with chronic conditions rose from 9.8% to 13.8% in this population. Compared with 2008, the adjusted odds of having a chronic condition in 2013 were 1.5 (95% confidence interval, 1.3–1.6). Despite rising chronic condition prevalence, average outpatient cost, visit frequency, and days between appointments did not grow significantly over time. However, total outpatient cost and visits increased for chronic condition patients during the study period, and total days between appointments decreased. Throughout the study period asthma was consistently a highly prevalent chronic condition, affecting approximately 6% of the population each year. The most growth in prevalence occurred among patients with diagnosed ADD (3.2% to 6.8%) and depression (0.3% to 1.0%). By 2013 ADD was the most prevalent chronic condition among pediatric patients.

## Discussion

Our study demonstrates that prevalence of MCCs among pediatric patients is increasing along with associated health care costs and office visits, whereas time between outpatient appointments is decreasing. We found that the top 5 most prevalent chronic conditions accounted for over 30% of outpatient costs attributable to pediatric patients.

An inpatient study by Zhong and colleagues found that more than 40% of pediatric patients had 1 chronic condition, whereas 17% had MCCs (7). Our investigation yielded much lower estimates because we studied an outpatient pediatric population; outpatients are less likely than hospitalized patients to have chronic diseases. However, as in our analysis, Zhong et al found that health care costs increased substantially with increasing numbers of chronic conditions (7). MCCs among pediatric patients have also been associated with increased use of in-hospital resources (8). Although we did not study costs associated with specific outpatient procedures, as Simon et al did (8), we found that pediatric MCC patients had significantly more outpatient visits than patients with no chronic conditions.

Chronic conditions can affect a child’s emotional, physical, and social development and often have lasting health and health care consequences (9,10). Unlike adults with MCCs, children with MCCs face unique challenges to treatment adherence, disease acceptance, lifestyle modification, care coordination, reduction of exposure to chronic condition risk factors, and transitioning to adult health care settings (11). Recently published MCC research does not identify children as a population requiring future investigation (12,13). Given the adverse personal burden experienced by children with chronic conditions and the effect these conditions have on health care use and cost as demonstrated by this study, this population warrants more in-depth examination, especially given that these patients are likely to develop additional health problems and grow even more clinically complex as they age.

An important limitation of this study is that our results may not be applicable to children whose parents are unemployed. Our analysis captures data on pediatric patients whose parents have diverse occupations in terms of salary (eg, clinicians, laboratory technicians, clerical staff). However, it would be inappropriate to apply our results to uninsured, unemployed, or Medicaid populations. We know that people at the greatest risk for acquiring chronic conditions are those from low social and economic classes (14), who were not represented in our study. Thus, our results probably underestimate the prevalence and burden of MCCs among children in Southeastern, Michigan. Another limitation of this study is the use of ICD-9-CM codes to identify chronic conditions. The validity of diagnosis codes could have been improved if additional clinical or laboratory data had been available. Additionally, our results probably underestimate the prevalence of chronic conditions overall in this population because we did not identify additional chronic illnesses, including cerebral palsy, cystic fibrosis, disorders of malnutrition, consequences of low birth weight, or congenital defects. The greater the number of chronic conditions considered in MCC studies, the higher the MCC prevalence found (15). Lastly, important covariates associated with chronic conditions, such as socioeconomic status, ethnicity/race, and exposure to tobacco smoke, were not available for adjusted analyses, which may have resulted in overestimation of regression results.

Our investigation helps to demonstrate the importance of studying MCCs among pediatric patients, because MCCs are growing in prevalence and are associated with increased health care use and cost. Asthma, ADD, depression, and diabetes are prevalent and costly chronic conditions among children and are appropriate targets for intervention.
